# Developing and using ontologies in behavioural science: addressing issues raised

**DOI:** 10.12688/wellcomeopenres.18211.2

**Published:** 2023-07-25

**Authors:** Susan Michie, Janna Hastings, Marie Johnston, Nelli Hankonen, Alison J. Wright, Robert West

**Affiliations:** 1 Centre for Behaviour Change, University College London, London, UK; 2 Faculty of Medicine, University of Zurich, Zurich, Switzerland; 3 School of Medicine, University of St. Gallen, St Gallen, Switzerland; 4 Aberdeen Health Psychology Group, University of Aberdeen, Aberdeen, UK; 5 Faculty of Social Sciences, Tampere University, Helsinki, Finland; 6 Research Department of Epidemiology & Public Health, University College London, London, UK

**Keywords:** ontology, behavioural science

## Abstract

Ontologies are ways of representing aspects of the world in terms of uniquely defined classes of ‘entities’ and relationships between them. They are widely used in biological science, data science and commerce because they provide clarity, consistency, and the ability to link information and data from different sources. Ontologies offer great promise as representational systems in behavioural science and could revolutionise descriptions of studies and findings, and the expression of models and theories.

This paper discusses issues that have been raised about using ontologies in behavioural science and how these can be addressed. The issues arise partly from the way that ontologies represent information, which can be perceived as reductionist or simplistic, and partly from issues to do with their implementation. However, despite the simplicity of their structure, ontologies can represent complex entities that change over time, as well as their inter-relationships and highly nuanced information about them. Nevertheless, ontologies are only one of many ways of representing information and it is important to recognise when other forms are more efficient.

With regard to implementation, it is important to build ontologies with involvement from the communities who will be using them. Far from constraining intellectual creativity, ontologies that are broadly-based can facilitate expression of nuance, comparison of findings and integration of different approaches and theories. Maintaining and updating ontologies remain significant challenges but can be achieved through establishing and coordinating communities of practice.

## Disclaimer

The views expressed in this article are those of the author(s). Publication in Wellcome Open Research does not imply endorsement by Wellcome.

## Introduction

*“Accuracy is, in every case, advantageous to beauty, and just reasoning to delicate sentiment. In vain would we exalt the one by depreciating the other.”* ( Hume, 1750, p.8)

Behavioural science is an interdisciplinary field of study whose aim is to understand and predict behaviour. It draws on many disciplines including psychology, sociology, anthropology, economics and neuroscience. It has helped to promote human health and wellbeing and will be very important in meeting the challenges facing humanity in the decades to come. However, cumulative scientific progress is being hampered by ambiguity, incoherence and inconsistency in the way that information about the domain is represented and reported. For example, reporting of behavioural interventions is typically less complete than for pharmacological interventions or other types of non-pharmacological interventions ( McCleary *et al.*, 2013). Moreover, a lack of shared terminology hinders evidence accumulation even when information is reported ( Michie & Johnston, 2017).

Ontologies, standardised representational frameworks providing a set of terms for the consistent description of data and information across disciplinary and research community boundaries ( Arp *et al.,* 2015), could go a long way to solving these problems ( National Academies of Sciences Engineering and Medicine, 2022). Indeed, recent applications of ontologies within the behavioural sciences include the Behaviour Change Intervention Ontology (BCIO) ( Michie *et al.*, 2021), the Addiction Ontology (AddictO) ( Hastings *et al.*, 2020) and the Mental Functioning suite of ontologies ( Larsen & Hastings, 2018). Ontologies offer great promise as representational systems in behavioural science and could radically improve descriptions of studies and findings, and the expression of models and theories. However, informal feedback during dissemination activities raised some issues about the potential limitations of ontologies in behavioural science. This paper describes and addresses the issues raised.

Ontologies represent information in the form of unique classes of ‘entities’ (e.g., processes and objects and their attributes) and the relationships between them ( Hastings, 2017). Each class has a label, a unique identifier ( McMurry *et al.*, 2017) and a definition. For example, in the Behaviour Change Intervention Ontology (BCIO) ( Michie *et al.*, 2021) the class that is labelled ‘individual human behaviour’ has the unique identifier BCIO:036000 (searchable via http://bciosearch.org) and is defined as ‘individual human activity that involves co-ordinated contraction of striated muscles controlled by the brain’. The definition, rather than the label, is the primary specification of a class’s meaning. Thus, ontologies enhance clarity and consistency by allowing definitions to be referred to directly through their associated identifiers (e.g. ‘BCIO:036000’) regardless of the label used to refer to them, thereby avoiding ambiguity or uncertainty as to what is being referred.

In ontologies, the properties of classes are given in each class’s definition. Definitions of classes in ontologies have a standard format, namely ‘A is a B that C’, where ‘A’ is the class being defined, ‘B’ is a parent class to which A belongs and ‘C’ describes a set of properties of A that distinguish it from other members of the same parent class ( Michie *et al*., 2022; Seppälä *et al*., 2017). For example, ‘individual human behaviour’ is defined as ‘A bodily process of a human that involves co-ordinated contraction of striated muscles controlled by the brain.’, where ‘bodily process’ is the parent class and ‘involves co-ordinated contraction of striated muscles controlled by the brain’ are the properties that distinguish this class from other bodily processes. The relationship between A and B is an example of the ‘subclass of’ relationship (also known as the ‘has parent class’ or ‘is a’ relationship) that creates the main hierarchical structure of an ontology. For example, ‘individual human behaviour’ is a subclass of ‘bodily process’. Where a ‘subclass of’ relationship exists, it implies that a class inherits all the properties of its parent class. Classes can also be further specified through explicitly specified relationships with other classes. For example, in the BCIO, ‘behaviour change intervention evaluation study finding’ is specified as being ‘output of’ a ‘behaviour change intervention evaluation study’.

Ontologies also allow users to reason logically using the relationships between classes. For example, the ‘subclass of’ relationship implies that a class inherits all the properties of *all* its parent classes. This allows an efficient representation in which each property only needs to be specified once at the right level of generality, and then users and computers are able to infer that it also applies for all the descendants of a given class. For example, knowing that communication behaviour (BCIO:036034) is classified as a subclass of inter-personal behaviour (BCIO:036025) allows the inference that communication behaviour involves an interaction between two or more people (from the definition of inter-personal behaviour) It also allows the inference that communication behaviour is ‘Individual human activity that involves co-ordinated contraction of striated muscles controlled by the brain’ (from the definition of ‘individual human behaviour,’ which is the parent of ‘inter-personal behaviour’). 

The clarity, consistency and facility for reasoning provided by ontologies make them extremely powerful tools when it comes to searching for information and linking items of information together. These are essential attributes for the conduct of science. Ontologies are widely used in biology, computer science and commerce. They provide a way of integrating information across studies, databases, models and disciplines.

In the course of the development and dissemination of the BCIO a number of issues were raised about the potential limitations of ontologies and potential adverse consequences of their use in fields of study such as behavioural science. The aim of this paper is to discuss and address these issues.

## Issues arising from the simplicity of the representational system

1. *Can ontologies represent different perspectives and nuances of ideas?*
  Some consider that because ontologies represent a positivist epistemology, they are not able to integrate different perspectives about the way the world is, nor represent complex and nuanced relationships. Ontologies consider that there is an objective world that can be described; they do not go beyond that and do not preclude different perspectives on that objective world. Rather, they aim to provide a framework within which knowledge can be captured in a way that clarifies differing perspectives, so that these can be explored in relation to each other. Further, by linking data arising from bodies of knowledge, ontologies are able to generate larger bodies of data representing heterogeneous knowledge. This, in turn, allows more complex analyses and predictions to novel scenarios that are not possible without this. In this way, ontologies can expand rather than reduce knowledge.
  Human experience can be expressed in forms that can’t be defined or necessarily related to other forms of representation, such as creative writing, visual art and music. Their purpose is often to evoke feelings of various kinds rather than to represent knowledge and where the latter is the purpose it is not in order to integrate with other forms of knowledge.
  When it comes to advancing knowledge through the scientific process, if ideas are considered too ‘complex and nuanced’ to define or to document in relation to others, they can’t be used in the scientific process. They would require ‘preprocessing’ to provide sufficient clarity to become part of the scientific endeavour.
2. *Can concepts of the kind used in behavioural science be defined?*
  Many classes employed in behavioural science are impossible to unambiguously define. This may be because of variations in usage (e.g. ‘adolescence’ covers age ranges that differ from one use to another) or inherent subjectivity (e.g. ‘craving’ which is a subjective experience that is not possible to specify entirely objectively). Many classes are also multifaceted and highly nuanced (e.g. ‘stress’ which involves a complex blend of physiological and subjective aspects).
  There are two parts to the response to this concern. One is that class definitions can represent any level of ambiguity, subjectivity and complexity that is desired. Moreover, they can be updated and evolve as our understanding evolves. Thus ontologies represent our current understanding of reality. If that understanding involves classes with fuzzy boundaries, subjective experience or a high degree of complexity, ontological class definitions can reflect that. Constructing good ontological definitions should always involve seeking the least ambiguity and subjectivity possible, but if the subject matter is not conducive to that, we have to acknowledge this but make it clear that we are doing so and what this implies.
  For example, with classes such as ‘adolescence’ with variable boundaries in practical use, we can express the variability in the definition and include a comment that when the class is used, it is important to operationalise it with a clear specification of the age range being used in that instance. With classes that may refer to subjective experiences such as ‘craving’, we can make clear in the definition that it is a type of subjective experience and what type of experience it is. If it is felt that there is a need for a class to cover the physiological processes involved in that experience, we can create a separate class for this which we might call ‘physiological craving’. Ontologies enable us to make these kinds of distinctions which are scientifically necessary but often overlooked.
  With some complex classes such as behaviour change techniques ( Michie *et al.*, 2013), it is important to accompany the definitions with elaborations, examples and even potentially training aids, to achieve the maximum possible level of reliability and consistency in using them ( Michie *et al.*, 2015). This would be the case even if they were not part of an ontology. Even if full objective consistency cannot be attained, greater clarity and consistency must always be better in science than lower clarity and consistency.
  The second part of the response is that in behavioural science, there are a large number of classes that can and should be defined objectively and precisely but which currently are not. Ontologies are well suited to do this. For example, behavioural outcomes in evaluations of interventions are rarely fully defined and this can make interpretation of findings and comparison across studies problematic. Behavioural outcomes can, and arguably should, be defined by combining ontological classes to create fully specified objectively defined expressions. For example, in tobacco use cessation studies outcome expressions need to include a relatively large number of components, each of which is itself a class in the ontology with a definition, such as:
  • ‘Tobacco use’ as the class of behaviour,
  • Negation of this behaviour to denote abstinence,
  • A reference point for timings being covered (e.g., ‘target quit date’),
  • Time points for the start and end of the assessment period (e.g., ‘2-weeks post’ to ‘26 weeks post’),
  • Method of assessment (e.g., ‘self-report’ at ‘26-week follow-up’, assessed by ‘in-person closed response oral question’, confirmed by ‘saliva cotinine concentration’ with ‘confirmation threshold’ <10ng/ml, with ‘assessor’ ‘blind to study group’, and ‘missing equals non-abstinence’ ‘missing value imputation’).
3. *Can complex relationships involving dynamic interactions between multiple entities be captured by dyadic relationships?*
  Ontologies expressed in the most widely used ontology language, OWL (the Web Ontology Language) ( Hitzler *et al.*, 2012), can only represent dyadic relationships: i.e., relationships between pairs of classes, as the OWL language does not allow relationships between more than two classes. However, complex causal relationships are common in behavioural science. For example, the desirability of a behaviour can be modelled as (at least sometimes) a function of the perceived desirability of the possible outcomes of the behaviour weighted by the perceived likelihood of their occurring as a result of the behaviour. This is a complex function involving interactions between multiple entities.
  The way that ontologies can handle complex relationships involving multiple classes is to break them down into pairwise relationships. This is not the most concise way of representing such relationships, but it is accurate and works for any level of complexity. To achieve an ontology handling complex relationships involving multiple classes, the ontology can include relationships themselves as classes, because ontological classes can represent any type of entity. Therefore, a class can be stated to have a causal relationship with another relationship, indicating a moderator relationship. Multiple examples of this are shown in the ‘Ontology-Based Modelling System’ (OBMS) set out by Hale *et al.* (2020). For example, in Change Theory ( https://theory-database.appspot.com/theory/6), ‘driving forces’ and ‘restraining forces’ both moderate the transition relationship which holds between ‘quasi-stationary equilibrium’ and ‘unfreezing’, and also the subsequent transition relationship between ‘unfreezing’ and ‘moving’. Relationships can be quantitative and involve any mathematical or statistical function, including linear and non-linear relationships.
  It is important to note that ontologies contain relationships that always obtain between entities, not relationships that only hold in certain instances. To give a simple example, a behaviour change intervention designer might include two behaviour change techniques (BCTs) ( Michie *et al.,* 2013) in an intervention, because they believe the first BCT will only change behaviour if the second BCT is also applied. However, in the general case these BCTs could be applied separately in other interventions. If the impact of the first BCT on behaviour is not always dependent on the second BCT, then an ontology should not contain this moderator relationship between the two BCTs. Therefore, the fact that certain complex relationships between variables are not specified in an ontology does not mean that the ontology implies that such relationships cannot exist in particular instances.
  Ontologies can promote clearer and more coherent modelling of causal relationships than is often seen in behavioural science. If ontologies are built using an upper-level ontology such as Basic Formal Ontology (BFO), as the BCIO is, they make a fundamental distinction between different kinds of entities that stand in different causal relationships with each other. Any BFO class (objects, processes, attributes etc) can in principle stand in a causal relationship with any other class. Thus, the presence of a stop sign (object) can cause drivers to stop (process); the slope of a hill (attribute) can cause cyclists to cycle slowly (process); and a police officer flagging down a motorist (process) can cause that motorist to pull over (process).
  Having said this, ontologies are not the optimal way of representing *all* information in behavioural science – only for definitional information about classes of entities. Statistical models, equations and algorithms are also crucial, and natural language will continue to dominate the communication of information.
4. *Can ontologies capture information at multiple levels, e.g., individual, group, society?*
  Ontologies are well suited to representing information at multiple levels and linking those levels together. Individuals are entities, as are groups and societies. Individuals can be linked to groups through relationships such as ‘member of’, and groups can be related to larger social entities in the same way. Groups are aggregates of people and can be ascribed properties just as individuals can. In some cases, these might be statistically related to the properties of individuals (e.g., ‘has mean age’) while in other cases they may only apply at the group level (e.g., ‘has group norm’).
5. *Do ontologies stifle creativity and diversity of views?*
  One issue raised is that by seeking to promote agreed definitions of classes, ontologies could restrict the freedom to arrive at different definitions that is essential for many aspects of innovative progress in scientific research. However, ontological definitions are not the same as dictionary definitions. Dictionary definitions are statements of the conventional meaning of words or phrases as used in language. Their purpose is to explicate the meaning(s) of terminology, which may differ from context to context. Ontological definitions are different, in that they aim to uniquely pick out a specific entity or class of entity (a specific type of thing) regardless of how that entity is usually referred to in language ( Michie *et al.,* 2022). Moreover, in ontologies, classes each have a unique *identifier* (distinct from the label) so that they can be clearly referenced. Classes are then given a *label* so that people can refer to them easily. Other people are not precluded from using that *label* to refer to something with a different definition in a different ontology or other classification system, as long as everyone is clear what the label refers to – the unique identifier and the definition provide disambiguation ( Michie *et al.*, 2022). An important aspect of ontologies is that when identifying whether something is an example of an ontological entity, it is important to be guided by the entity’s definition rather than its label.
  It is preferable, from a practical point of view, for a scientific community to use the same labels to refer to the same things. However, in a field of study such as behavioural science it is understandable that different members of the community may prefer to use labels in different ways. At present, the usage of the same labels in different ways is done without making it clear that this is what is being done, so it can be difficult in practice to determine the precise entities that are intended by specific labels being used. Ontologies provide clarity by assigning unique identifiers to every semantically distinguishable class, which can be used alongside the label to clarify the intended definition of a class. This is also helpful for the opposite problem: people using different labels for what may be intended to be the same entity. For example, while psychologists commonly refer to behaviour, sociologists favour the term social practices and anthropologists often refer to habitus. Whilst these words all have different nuances and implications, they also share an essence of meaning, which can be clarified by ontological definitions.
  An example of using ontological definitions to achieve clarity is the contested term ‘addiction’ ( Kelly *et al.*, 2022). A consensus can be achieved that there is a class that can be defined along the lines of ‘A disposition to experience strong motivation to engage in a behaviour to an extent that can override self-conscious attempts at restraint’. This class can be given a unique ID, and perhaps the label ‘compulsion’. However, others may want to refer to the entity with this definition as ‘addiction’. Other researchers may be interested in social roles and communities and the ways that social inclusion and reward act to create distinct substance usage patterns of behaviour, which they may also wish to refer to as ‘addiction’ or ‘compulsion’. In that case, they could define such a class and give it an ID and a label. The extent to which any specific class is used will depend on how useful the broader community of researchers and practitioners finds them.
  To avoid confusion, class labels should be unique *within an ontology*. Ideally, primary labels for classes in ontologies should be constructed in a way that makes them interpretable without knowing the context. Labels used in different ontologies for classes with different definitions are then disambiguated by stating the ontology that they come from in their unique identifier. The specifier of the ontology is known as the ‘namespace’. Thus for the BCIO class, ‘individual human behaviour,’ with the unique identifier ‘BCIO:036000’, ‘BCIO’ is the namespace, telling the reader that the class comes from the Behaviour Change Intervention Ontology, and the reader can assume that this label ‘individual human behaviour’ is unique in the BCIO. Classes may however be associated with multiple non-unique synonyms in order to reflect broader usage patterns that are ambiguous.
6. *Are ontologies necessarily reductionist?*
  Reductionism is an approach to analysing complex phenomena that breaks the phenomena down into their component parts. The limitation of this approach is that there are occasions when it fails to address the emergent properties of complex systems.
  Ontologies can be reductionist but they need not be. There are ontologies that only include simple atomic components of the system, for example, the chemistry ontology ChEBI ( Degtyarenko *et al.*, 2008) largely encompasses individual small molecular entities, but as has already been noted, ontologies can include classes at multiple levels – ChEBI includes some bulk substances and mixtures, and the BCIO’s Population module includes both individual personal attributes and aggregate population attributes. Therefore, ontologies can include classes at all levels of a complex system. Importantly, ontologies can represent relationships between classes at different levels, including those that involve self-reference, complex feedback loops or changes over time, such as human social identities.
  For example, when attempting to represent behavioural aspects of combating the Covid-19 pandemic, an ontology can and should include classes relating to individuals, behaviours, family units, peer groups, sociodemographic groups, health agencies, companies and local and national governments and fully capture any set of relationships that exist within and between them, such as social influence processes and dynamic interpersonal interactions.
  One issue raised by the community of researchers and practitioners engaged in developing and evaluating behaviour change interventions is that classification systems, such as ontologies, may inhibit creativity in intervention development by pre-specifying too much about the intervention through the associated definitions. However, ontologies such as the Behaviour Change Intervention Ontology can be thought of as simply providing a set of potential ingredients for interventions. The ontology does not restrict the ways in which those ingredients can be combined. Moreover, ontologies are not static: if new ‘ingredients’, or important aspects of interventions, are subsequently identified, they can be added to the ontology.

## Issues raised relating to the implementation of ontologies

1. *Does the use of ontologies require specialist training?*
  Although access to ontology content and definitions is easily available to anyone with an internet connection, ontologies do nevertheless require knowledge and skills to use within research or practice, and in particular specialist training is needed to develop or extend ontologies. At present, there is very limited expertise within the behavioural and social sciences and it will be necessary to expand and extend this. As with other methodologies, such as statistical or qualitative analysis, different types of users will have differing levels of expertise.
  There is a need for highly specialised ontologists, with a computer science or other logic-heavy background, to take a lead in the technical aspects supporting the development and use of ontologies and verify their formal logical structures. There will also be a need for domain experts with a strong grounding in ontologies to take part in developing and updating their content, and to create guides that non-experts can use when contributing to or using ontologies.
  Most behavioural scientists working with ontologies will use ones that have already been developed. The most common use will be ensuring that the constructs that they use in their work are drawn from an ontology, or where no such construct can be found within an existing ontology, proposing a new construct through some kind of user interface that is provided with the ontology.
2. *How can ontology developers motivate and enable people to use ontologies?*
  Researchers and practitioners will use ontologies if they see it as in their or their field’s interests to do so. Initially, the early adopters will be people who buy into the benefits in principle and find the experience rewarding. However, for more widespread adoption, promoters of ontologies will have to pay heed to simple behavioural science principles, making use of ontologies: normal, easy, attractive and routine ( West *et al.*, 2020). This will probably require the gatekeepers of science (primarily funders, journals and organisations that undertake high profile systematic reviews) to support, and ultimately to require, their use.
  Crucially ontologies need to be developed in partnership with people who will be using them ( Norris *et al.*, 2021). The process of ontology development needs to meet the needs of users. This means that ontologies must reflect rather than challenge common usage of terms. This can be problematic when that usage is inconsistent or confused. In those cases, ontology development and implementation requires the community of users to be supported to build their knowledge and skills towards attention to, thinking and expression of finer points of distinction and subtle differences in meanings that ultimately enable the ontology to reflect a consensus, accurate and coherent whole that nevertheless is true to the multiplicity of perspectives.
3. *How can ontologies be maintained and updated?*
  Ontologies need to be maintained. They have to be held in one or more online repositories and the software tools that enable people to use them need to be made available. Ontologies also need to be continually developed, updated and expanded as fields advance and users bring new insights. All of this requires resources and organisations of some kind to take responsibility for the process.
  This can be very challenging. Funders have thus far not typically been supportive of the kind of long-term infrastructural funding needed to provide a stable home for ontologies. Instead, where ontologies have been successfully maintained and updated, they have either served the needs of particular organisations or consortia who have been willing to put the necessary resource into them, or been sustained collaboratively through multiple users each budgeted in projects a small amount to, essentially, buy ontology services.
  Most likely, maintaining and updating ontologies will require establishing vibrant communities of practice in which most of the people working in a particular domain are invested in maintaining and developing the relevant ontologies. It is also through the development of such critical mass within communities that alternative approaches to more sustainable funding for the future can be actualised.
4. *Is there a risk that ontologies will be taken over by powerful groups or vested interests?*
  Science is a social enterprise and has always been shaped by power relations among those involved and the context in which science is funded and promoted. These power relations typically have the effect that certain perspectives and viewpoints tend to dominate. Ontologies promote transparency and bring conceptualisation into the open. They are explicitly linked to a community of developers and maintainers and use open-source techniques to track their evolution and progress over time. For example, the BCIO and AddictO are both hosted on the GitHub online platform. GitHub provides an open, sustainable and low-cost portal for the scientific community to suggest and discuss potential changes. It includes an issue tracker, allowing feedback to be submitted which can be openly replied to, discussed and, if appropriate, addressed in subsequent releases of the ontology. GitHub also has in-built mechanisms for tracking releases and versioning, so anyone can see how the ontology is updated in response to feedback. Therefore, far from promoting hegemony on the part of a dominant group, ontologies can expose hegemonies where these occur and offer the prospect for those who are not part of a dominant perspective to contribute to the development of a shared ontology for the domain. A key additional benefit of ontologies is that they can be linked within and across scientific disciplines, thus making more efficient the accumulation and translation of knowledge across boundaries that at present are enforced by academic divisions. The near-universal access to the internet means that there is an infrastructure that can support access to new perspectives.

## Conclusions

Ontologies offer promise as a representational system in behavioural science and could revolutionise descriptions of studies and findings, and the expression of models and theories. Ontologies offer much greater clarity and consistency than the unstructured scientific communication practices that dominate at present, and herald a new era in evidence searching and synthesis. However, there are challenges that need to be overcome for them to fulfil their potential, and there are pitfalls in their implementation. We need to educate and train behavioural and social scientists in the use of ontologies and build a cadre of ontology experts, just as we have cadres of people who are experts in designing randomised trials or undertaking complex qualitative analyses. Ultimately, we will need a large community of practice that is self-sustaining.

## Data Availability

No data are associated with this article.
