# Supplementary material for: Developing and using ontologies in behavioural science: addressing issues raised
Source: Wellcome Open Res. 2023 Jul 25;7:222. Originally published 2022 Aug 30. [Version 2] doi: 10.12688/wellcomeopenres.18211.2 (PMC11109559; doi:10.12688/wellcomeopenres.18211.2)
Supplement: Peer review history

# Reviewer report: Reviewer response for version 2

**DOI:** 10.21956/wellcomeopenres.21913.r77364

**Published:** 2024-05-07

**Authors:** Bryan A Kutner

**Affiliations:** 1 Albert Einstein College of Medicine, New York, USA

This article summarizes and responds to challenges raised during the course of the development and dissemination of ontologies for the behavioral sciences. The authors and previous reviewers raise stimulating points of tension by documenting arguments against and for the use of ontologies in the behavioral sciences. This furthers our ability to discern areas for scientific growth. I see only a few additional opportunities that I hope may strengthen the article.

 I wonder if there’s a case to say that ontologies are particularly important right now, because with generative AI we are on the cusp of being able to expedite the synthesis of behavioral science only if we train these processes to grasp behavioral constructs and their interrelations. There is so much content in the behavioral sciences, accumulating exponentially in ways that outpace any single person or research group’s ability to synthesize knowledge across studies. The use of ontologies in conjunction with generative AI, while not without risks, could advance our accumulation of knowledge about what does and does not work in ways that may not be as feasible by other means.

 This question also raises for me a concern about the ways human beings and systems that produce and disseminate scientific knowledge bias the categorization within ontologies. For example, in what ways does publication bias constrain our understanding of the interrelations of constructs? How do we solve that issue going forward? Given these biases, how do ontologies contribute to the accumulation of knowledge in an errant direction and how can they contribute to correcting the scientific endeavor, if possible?

 I appreciate the authors’ call to action that the perfect should not be the enemy of the good in behavioral science: “Even if full objective consistency cannot be attained, greater clarity and consistency must always be better in science than lower clarity and consistency.” Additionally, I appreciate that the authors address the exertion of power in science and how the use of ontologies, if democratized to the extent possible, may serve as opportunities to challenge hegemony. I was also very pleased to see the authors address the need for specialists to take the lead in technical aspects of supporting the development and use of ontologies. Back to the question about hegemony, what is the ‘governing’ body that determines revisions or additions to a given ontology? Who inherits those rights for a given ontology, or does an ontology exist without ‘copyright’?

 In the example provided about “addiction” and “compulsion”, I think that the point of choice about which ontology to reference could be made slightly clearer. A diversity of ontologies exist and behavioral scientists who want to specify “compulsion” as something different than “addiction”, for example, may choose to mix and match from the overall set of ontologies to specify nuanced differences between ontologies. Is this appropriate or should scientists try to stick with a single ontology as a cohesive whole representation of the world of behavior sciences?

 One specific request: I would benefit from a clearer understanding of “preprocessing” with an example and a window into what this preprocessing might entail. To the authors’ point about the benefits of ontologies, better for me to really understand that as a reader than to move forward with an idiosyncratic understanding that doesn’t actually match what is intended.

 There might be a typo toward the end: “…subtle differences in meanings that ultimately enable the ontology to reflect a consensus, **AN** accurate and coherent whole that nevertheless is true to the multiplicity of perspectives.”

 This was a terrifically stimulating read – thank you for the contribution.

Does the article adequately reference differing views and opinions?

Yes

Are all factual statements correct, and are statements and arguments made adequately supported by citations?

Yes

Is the Open Letter written in accessible language?

Yes

Where applicable, are recommendations and next steps explained clearly for others to follow?

Yes

Is the rationale for the Open Letter provided in sufficient detail?

Yes

Reviewer Expertise:

Behavioral science, implementation science, HIV/AIDS, stigma intervention

I confirm that I have read this submission and believe that I have an appropriate level of expertise to confirm that it is of an acceptable scientific standard.

# Reviewer report: Reviewer response for version 2

**DOI:** 10.21956/wellcomeopenres.21913.r63699

**Published:** 2023-07-26

**Authors:** Linda M. Collins

**Affiliations:** 1 School of Global Public Health, New York University, New York, New York, USA

I do not have any additional comments. The revisions enhance the article.

Does the article adequately reference differing views and opinions?

Yes

Are all factual statements correct, and are statements and arguments made adequately supported by citations?

Yes

Is the Open Letter written in accessible language?

Partly

Where applicable, are recommendations and next steps explained clearly for others to follow?

Yes

Is the rationale for the Open Letter provided in sufficient detail?

Yes

Reviewer Expertise:

Behavioral science research methods

I confirm that I have read this submission and believe that I have an appropriate level of expertise to confirm that it is of an acceptable scientific standard.

# Reviewer report: Reviewer response for version 1

**DOI:** 10.21956/wellcomeopenres.20190.r56905

**Published:** 2023-05-16

**Authors:** Linda M. Collins

**Affiliations:** 1 School of Global Public Health, New York University, New York, New York, USA

This Open Letter responds to a variety of questions that have been asked about, and criticisms that have been leveled at, ontologies developed for use in behavioral science.  Ontologies seem to make some people uncomfortable. This letter does a good job of articulating the potential reasons why and responding to them.  I do want to recommend trying to make the following sections a bit easier to read: the two paragraphs at the top of the right-hand column on p. 3, and the last paragraph on p. 6.

 Ontologies, such as the Behaviour Change Intervention Ontology (BCIO), are essentially no more and no less than tools for facilitating scientific discourse.  In other words, they are descriptive, not prescriptive.  They do not set the scientific agenda, but they make an agenda easier to carry out once it has been set.  One important way ontologies facilitate scientific discourse is by making computer searches easier to carry out and more accurate.  Ontologies are not written in stone; on the contrary, they can and should evolve continually as scientific thinking evolves.

 The letter mentions that some people believe ontologies are overly reductionistic and therefore have an inhibiting effect on creativity.  However, I find that the increased clarity lent by ontologies is stimulating, helps to open up new possibilities, and helps make thinking more productive.  It also helps to highlight exactly where the differences lie among competing perspectives.

 In my view, it is important to remember that ontologies do not have to be perfect to play a valuable role in behavioral science.  They merely have to be better than the alternative, which is fewer clear definitions of terms, less consistency across areas within behavioral science, and little basis for arriving at consensus.  As the authors say, "Even if full objective consistency cannot be attained, greater clarity and consistency must always be better in science than lower clarity and consistency."

Does the article adequately reference differing views and opinions?

Yes

Are all factual statements correct, and are statements and arguments made adequately supported by citations?

Yes

Is the Open Letter written in accessible language?

Partly

Where applicable, are recommendations and next steps explained clearly for others to follow?

Yes

Is the rationale for the Open Letter provided in sufficient detail?

Yes

Reviewer Expertise:

Behavioral science research methods

I confirm that I have read this submission and believe that I have an appropriate level of expertise to confirm that it is of an acceptable scientific standard.

# Response

**Published:** 2023-07-21

**Authors:** Susan Michie

**Affiliations:** University College London, UK

This Open Letter responds to a variety of questions that have been asked about, and criticisms that have been leveled at, ontologies developed for use in behavioral science.  Ontologies seem to make some people uncomfortable. This letter does a good job of articulating the potential reasons why and responding to them.

- *Response: We thank the reviewer for the positive feedback about this paper. All comments made by the reviewer have been addressed.*

I do want to recommend trying to make the following sections a bit easier to read: the two paragraphs at the top of the right-hand column on p. 3, and the last paragraph on p. 6. 

- *Response: For ease of reading and as suggested by the reviewer, we have revised these three paragraphs. We hope this is clearer now.*

Ontologies, such as the Behaviour Change Intervention Ontology (BCIO), are essentially no more and no less than tools for facilitating scientific discourse.  In other words, they are descriptive, not prescriptive.  They do not set the scientific agenda, but they make an agenda easier to carry out once it has been set.  One important way ontologies facilitate scientific discourse is by making computer searches easier to carry out and more accurate.  Ontologies are not written in stone; on the contrary, they can and should evolve continually as scientific thinking evolves. The letter mentions that some people believe ontologies are overly reductionistic and therefore have an inhibiting effect on creativity.  However, I find that the increased clarity lent by ontologies is stimulating, helps to open up new possibilities, and helps make thinking more productive.  It also helps to highlight exactly where the differences lie among competing perspectives. In my view, it is important to remember that ontologies do not have to be perfect to play a valuable role in behavioral science.  They merely have to be better than the alternative, which is fewer clear definitions of terms, less consistency across areas within behavioral science, and little basis for arriving at consensus.  As the authors say, "Even if full objective consistency cannot be attained, greater clarity and consistency must always be better in science than lower clarity and consistency." 

- *Response: We thank the reviewer for their insightful reflections on the open letter and for their positive feedback.*

# Reviewer report: Reviewer response for version 1

**DOI:** 10.21956/wellcomeopenres.20190.r55005

**Published:** 2023-03-16

**Authors:** Stefan Kaufman

**Affiliations:** 1 BehaviourWorks Australia, Monash Sustainable Development Institute, Monash University, Melbourne, Vic, Australia

This paper outlines key issues and implementation challenges raised by attempting to apply standardised representational frameworks (Ontologies) to behavioural science knowledge and evidence on - for example - reviewing evidence of efficacy of interventions. 

 It does a very good job of communicating a relatively abstract concept, and outlines a range of cogent criticisms, concerns and implementation challenges. To the extent that it fails to represent differing views and opinions, the analysis takes for granted a positivist epistemology, and doesn't seriously consider whether and how knowledge and evidence on behavioural phenomena from other perspectives might potentially be integrated, or excluded, from the concept of ontologies as explored in this review.  For example, researchers exploring habitus or practices have a lot to share on day to day activities, and what influences them, but would likely object to having to reduce their knowledge into pair-wise relationships and super-categories, and may struggle to match their knowledge constructs to the required input variables.  

 A related concern is that while the paper acknowledges the difficulty of reflecting complex relationships, it doesn't directly address the practical implications of this. In short, as the authors acknowledge - concepts and phenomena that are relatively simple are also relatively easy and efficient to represent in the proposed framework. Those that are not, could be represented, but require considerably more time and effort to include. Conceivably, we could anticipate an 'un-natural' selection of evidence and knowledge that had relatively low friction being over represented in the proposed framework, whereas more complex and nuanced knowledge would be under represented or not at all, unless resources, time and effort were directed to capture the difficult material. 

 This point also speaks to the implementation challenge 4 - the risk of capture by powerful or vested interests. To the extent that some phenomena (individual, positivist knowledge) is well represented, and more complicated, interpersonal and communal knowledge is not, the proposed framework could easily end up reproducing relatively individualist, libertarian analyses of complex problems, and not representing more holistic and communitarian analyses that might also implicate powerful actors like governments and corporations more directly. Unless pro-social funders like governments,  philanthropies and progressive businesses choose to  correct the imbalance. At present, in 2023, arguably relatively more funding and support is directed at the former than the latter.

Does the article adequately reference differing views and opinions?

Partly

Are all factual statements correct, and are statements and arguments made adequately supported by citations?

Yes

Is the Open Letter written in accessible language?

Yes

Where applicable, are recommendations and next steps explained clearly for others to follow?

Partly

Is the rationale for the Open Letter provided in sufficient detail?

Yes

Reviewer Expertise:

I apply the lens of behavioural change to understanding and accelerating urgent sustainability transitions required to maintain humanity with key planetary boundaries.

I confirm that I have read this submission and believe that I have an appropriate level of expertise to confirm that it is of an acceptable scientific standard, however I have significant reservations, as outlined above.

# Response

**Published:** 2023-07-21

**Authors:** Susan Michie

**Affiliations:** University College London, UK

This paper outlines key issues and implementation challenges raised by attempting to apply standardised representational frameworks (Ontologies) to behavioural science knowledge and evidence on - for example - reviewing evidence of efficacy of interventions. It does a very good job of communicating a relatively abstract concept, and outlines a range of cogent criticisms, concerns and implementation challenges.

- *Response: We thank the reviewer for the positive feedback about this paper. All comments made by the reviewer have been addressed.*

To the extent that it fails to represent differing views and opinions, the analysis takes for granted a positivist epistemology, and doesn't seriously consider whether and how knowledge and evidence on behavioural phenomena from other perspectives might potentially be integrated, or excluded, from the concept of ontologies as explored in this review.  For example, researchers exploring habitus or practices have a lot to share on day to day activities, and what influences them, but would likely object to having to reduce their knowledge into pair-wise relationships and super-categories, and may struggle to match their knowledge constructs to the required input variables.  A related concern is that while the paper acknowledges the difficulty of reflecting complex relationships, it doesn't directly address the practical implications of this. In short, as the authors acknowledge - concepts and phenomena that are relatively simple are also relatively easy and efficient to represent in the proposed framework. Those that are not, could be represented, but require considerably more time and effort to include. Conceivably, we could anticipate an 'un-natural' selection of evidence and knowledge that had relatively low friction being over represented in the proposed framework, whereas more complex and nuanced knowledge would be under represented or not at all, unless resources, time and effort were directed to capture the difficult material. This point also speaks to the implementation challenge 4 - the risk of capture by powerful or vested interests. To the extent that some phenomena (individual, positivist knowledge) is well represented, and more complicated, interpersonal and communal knowledge is not, the proposed framework could easily end up reproducing relatively individualist, libertarian analyses of complex problems, and not representing more holistic and communitarian analyses that might also implicate powerful actors like governments and corporations more directly. Unless pro-social funders like governments,  philanthropies and progressive businesses choose to  correct the imbalance. At present, in 2023, arguably relatively more funding and support is directed at the former than the latter.

- *Response: We thank the reviewer for raising these important points. We have now addressed and included this as a new issue in the section “Issues arising from the simplicity of the representational system” as follows:* *“1. Can ontologies represent different perspectives and nuances of ideas?* *Some consider that because ontologies represent a positivist epistemology, they are not able to integrate different perspectives about the way the world is, nor represent complex and nuanced relationships. *
* Ontologies consider that there is an objective world that can be described; they do not go beyond that and do not preclude different perspectives on that objective world. Rather, they aim to provide a framework within which knowledge can be captured in a way that clarifies differing perspectives, so that these can be explored in relation to each other. *
* Further, by linking data arising from bodies of knowledge, ontologies are able to generate larger bodies of data representing heterogeneous knowledge. This, in turn, allows more complex analyses and predictions to novel scenarios that are not possible without this. In this way, ontologies can expand rather than reduce knowledge.* *Human experience can be expressed in forms that can’t be defined or necessarily related to other forms of representation, such as creative writing, visual art and music. *
* Their purpose is often to evoke feelings of various kinds rather than to represent knowledge and where the latter is the purpose it is not in order to integrate with other forms of knowledge.* *When it comes to advancing knowledge through the scientific process, if ideas are considered too ‘complex and nuanced’ to define or to document in relation to others, they can’t be used in the scientific process. They would require ‘preprocessing’ to provide sufficient clarity to become part of the scientific endeavour.”*
- *The comments were very helpful. Thank you for the review*
